# A comparison of surgical outcomes between pre-and full-term patients with exotropia

**DOI:** 10.1371/journal.pone.0208848

**Published:** 2018-12-07

**Authors:** Eun Hye Jung, Young Suk Yu, Seong-Joon Kim

**Affiliations:** 1 Department of Ophthalmology, Chuncheon Sacred Heart Hospital, Chuncheon, Korea; 2 Department of Ophthalmology, Seoul National University College of Medicine, Seoul, Korea; 3 Department of Ophthalmology, Seoul National University Hospital, Seoul, Korea; Faculty of Medicine, Cairo University, EGYPT

## Abstract

**Purpose:**

To compare the surgical outcomes between pre- and full-term patients with exotropia and to examine the factors associated with surgical outcomes.

**Methods:**

This retrospective study included 48 pre- and 432 full-term patients with basic-type exotropia who underwent unilateral or bilateral lateral rectus muscle (ULR or BLR) recession. Preoperative characteristics and surgical outcomes were compared between the pre- and full-term infants. Additionally, factors affecting the surgical outcomes were evaluated in all patients.

**Results:**

The preoperative characteristics were significantly different between the pre- and full-term groups in terms of neurodevelopmental disabilities (p = 0.020). There were no significant differences between the pre- and full-term groups in terms of the success, overcorrection, and recurrence rates after the mean follow-up period of 34.6 ± 13.9 months (p = 0.697). The major cause of surgical failure was recurrence in both groups. Pre-term birth was not a risk factor for overcorrection and recurrence. However, regardless of the pre- or full-term birth status, the presence of neurodevelopmental disabilities significantly affected final overcorrection (p = 0.004).

**Conclusions:**

Pre-term patients with exotropia showed similar surgical outcomes to full-term controls. The presence of neurodevelopmental disabilities was a risk factor for final overcorrection.

## Introduction

Prematurity is known to have an increased rate of ophthalmological morbidities, such as myopia, amblyopia, strabismus, retinopathy of prematurity (ROP), and optic nerve abnormalities. [1, 2] Several studies reported that low birth weight, prematurity (<37 completed weeks gestation), ROP, and cerebral palsy are risk factors for strabismus. [3, 4] The increased number of premature and low birth-weight infants who survived led to an increased number of patients with strabismus. [3–5]

Ocular abnormalities, including refractive error and amblyopia in prematurity, were factors affecting the outcome of strabismus surgery, as well as risk factors for strabismus. [5–7] Pre-term birth is also related to various neurological or developmental abnormalities, including cerebral palsy, developmental delay/mental retardation, and seizure disorders, and damaging premature infant’s central nerve system affects ocular motor control and binocularity. [8, 9] Thus, pre-term children could show different surgical outcomes after strabismus surgery compared with full-term children.

Many studies reported the surgical outcomes of exotropia in pre-term or neurologically impaired children, [5, 7, 10–13] and many of these studies showed poor outcomes, especially high rates of overcorrection. [5, 12, 13] To date, there are a few studies about the surgical outcome of exotropia in patients with cerebral palsy or developmental delay. [12, 14–17] Furthermore, there are only a few studies on exotropia in pre-term patients with exotropia, and these involved small patient populations. [18] To our knowledge, no previous studies have compared the surgical outcomes of exotropic patients with pre- and full-term births. Therefore, this study aimed to evaluate the surgical motor and sensory outcomes between pre- and full-term patients who underwent unilateral or bilateral lateral rectus (ULR or BLR) recession for exotropia, and to investigate the factors related to surgical outcomes for exotropia using a multivariable analysis.

## Materials and methods

Approval to conduct this study was obtained from the Institutional Review Board of Seoul National University Hospital. The medical records of patients with basic-type exotropia, who underwent ULR or BLR recession when <18 years of age at Seoul National University Children’s Hospital between January 1, 2012 and December 31, 2015, were retrospectively reviewed.

We defined pre-term birth as birth before 37 weeks of gestational age, very pre-term birth as birth before 32 weeks of gestational age, and low birth weight as birth weight less than 2500 g. We included patients with neurological or developmental disabilities related to pre-term birth such as developmental delay (DD), cerebral palsy (CP), periventricular leukomalacia (PVL), intraventricular hemorrhage (IVH), and seizure disorders. [8] We excluded patients with genetic or chromosomal abnormalities, congenital abnormalities, and other ophthalmic, systemic, or neurological diseases except for disorders related to pre-term birth. Patients with paralytic or restrictive exotropia, A or V pattern, amblyopia (best corrected visual acuity of the worse eye ≤ 20/40 or no fix/follow), history of strabismus surgery, combined surgery for vertical strabismus, or a follow-up interval of <1 year were also excluded. Patients with associated strabismus (dissociated vertical deviation, oblique muscle overaction grade ≤ +1, or vertical deviation <8 prism diopters (PDs)) not requiring surgical correction were included.

### Preoperative examination

All patients underwent complete ophthalmological examinations before surgery, and the following data were recorded: gestational age, birth weight, sex, age at onset of deviation, age at diagnosis of exotropia, age at surgery, cycloplegic refraction, distance and near deviation angle, constancy of deviation, associated strabismus (dissociated vertical deviation, oblique muscle dysfunction, vertical deviation), presence of eye dominance, lateral incomitance (decrease in distant deviation of > 5 PD in a lateral gaze) [19], stereoacuity, and fusional status. Deviation was measured using the alternate prism and cover test at a distance (6 m) and close-up (33 cm) for the primary gaze, with appropriate spectacle correction when required. In cooperative patients, stereoacuity (near) and fusional status (distance and near) were determined using the Titmus stereoacuity and Worth 4-dot tests, respectively.

### Surgical technique and postoperative assessment

All surgeries were performed under general anesthesia by a single surgeon (S-JK) on the basis of the largest angle of preoperative deviation measured at distance or near. Table 1 provides the formula that was used for surgical procedures according to the surgeon’s experience. Patients with exotropia of <25 prism diopters both at distance and near underwent unilateral lateral rectus recession. The quantum of surgery was reduced by 0.5 mm per muscle in patients with neurodevelopmental disabilities and those <4 years of age at surgery to prevent overcorrection based on the personal experience of the surgeon and reports in previous studies. [5, 7, 10, 15, 20]

**Table 1:** Surgical dosage used for exotropia in this study.

Prism diopters	BLR recession, mm	ULR recession, mm
15	4	8.5
20	5.5	9.5
25	6	
30	6.5	
35	7.5	
40	8.5	
45	9.5	
50	9.5–10	

BLR = bilateral lateral rectus, ULR = unilateral lateral rectus

Postoperative assessments were made at 1 day, 1 week, and 3, 6, and 12 months after surgery, with an annual follow-up thereafter. Postoperative measurements of deviations were performed in the same manner as preoperative measurements. Surgical success was defined as esodeviation of <5 PD, orthotropia, or exodeviation of <10 PD at distance. Overcorrection was defined as esodeviation of ≥5 PD; recurrence, as exodeviation of ≥10 PD. Both overcorrection and recurrence were considered a surgical failure. We collected postoperative alignment data before reoperation. During the follow-up period in this study, no patient underwent reoperation for consecutive esotropia or recurrence after exotropia.

### Main outcome measures

Primary outcome measures included a comparison of surgical outcomes between pre- and full-term groups. Factors affecting surgical outcomes were also evaluated among all patients.

### Statistical analysis

All statistical analyses were performed with the Statistical Package for Social Sciences version 24.0 for Windows (SPSS Inc, Chicago, IL). A P-value of <0.05 was considered significant. The Pearson χ^2^, Fisher’s exact, independent t, and Mann-Whitney tests were used to compare patients’ characteristics and surgical outcomes. A linear mixed model was used to compare repeated measures of postoperative angle deviation at various time points between pre- and full-term patients. Cumulative probabilities of overcorrection and recurrence were compared between pre- and full-term using Kaplan-Meier survival analyses. Factors affecting recurrence rates over time were evaluated using the Cox proportional hazards regression model. Logistic regression analysis was used for factors affecting final overcorrection. Multivariable analysis that included factors determined by the univariable analysis to be statistically significant was performed.

## Results

### Patient demographics

The study included 480 patients with exotropia who underwent ULR or BLR recessions. Four hundred and thirty-two patients were full-term, and 48 patients were pre-term. The preoperative patient characteristics were not significantly different between the two groups except for the gestational age, birth weight, and neurodevelopmental disabilities (p = <0.001, <0.001, and 0.020, respectively) (Table 2). Among 48 pre-term patients, 9 were very pre-term. Among 432 full-term patients, 12 had neurodevelopmental disabilities (one with CP, one with epilepsy, one with IVH, and nine with developmental delay with no specific diagnosis), and among 48 pre-term patients, 5 had neurodevelopmental disabilities (one with microcephaly, one with epilepsy, one with PVL and IVL, two with developmental delay with no specific diagnosis). One patient had a history of laser treatment for retinopathy of prematurity. Mean postoperative follow-up times were 34.3 ± 13.8 months and 38.0 ± 15.1 months for the full- and pre-term groups, respectively, but this was not statistically significant (p = 0.081).

**Table 2:** Demographics and ocular characteristics of patients.

	Preterm group (N = 48)	Full-term group (N = 432)	P-value
Age, mo (range)			
onset of exotropia	31.1 ± 24.0 (6–86)	31.0 ± 23.3 (3–152)	0.966^a^
diagnosis of exotropia	49.6 ± 27.5 (14–112)	55.7 ± 29.3 (7–173)	0.169^a^
surgery	69.2 ± 26.7 (26–140)	76.0 ± 27.7 (16–185)	0.107^a^
Gestational age, wks (range)	34.3 ± 2.2 (27–36)	39.5 ± 1.1 (37–42)	< 0.001^a^
Birth weight (range)	2.3 ± 0.6 (0.8–3.8)	3.2 ± 0.4 (1.7–4.5)	< 0.001^a^
Neurodevelopmental disabilities	5 (10)	12 (3)	0.020^b^
Sex (M:F)	21:27	189:243	1.000^c^
Refractive errors in spherical equivalent, D (range)	-0.1 ± 1.9 (-6 to 6)	-0.1 ± 1.4 (-7 to 3)	0.996^a^
Type of exotropia (Intermittent:Constant)	34:14	312:119	0.819^c^
Preoperative deviation, PD (range)			
Distance	30.3 ± 7.9 (20–55)	30.2 ± 7.1 (14–55)	0.887^a^
Near	31.8 ± 8.8 (12–55)	31.6 ± 7.3 (14–60)	0.842^a^
Presence of eye dominance	14 (29)	179 (42)	0.093^c^
Lateral incomitance	13 (30)	122 (30)	0.999^c^
Associated vertical strabismus	11 (23)	137 (32)	0.207^c^
BLR:ULR recession procedure	37:11	355:77	0.387^c^
Amount of recession, mm (range)	12.6 ± 2.7 (9.0–20.0)	12.6 ± 2.6 (8.5–20.0)	0.820^a^
Postoperative follow-up, mo (range)	38.0 ± 15.1 (12–68)	34.3 ± 13.8 (12–72)	0.081^a^

BLR = bilateral lateral rectus,ULR = unilateral lateral rectus, D = diopter, PD = prism diopter

Data are presented as means ± standard deviations or as numbers (%).

^a^Independent t test

^b^Fisher’s exact test

^c^Pearson’s χ2 test

### Surgical outcomes between the pre- and full-term groups

**Fig 1:**
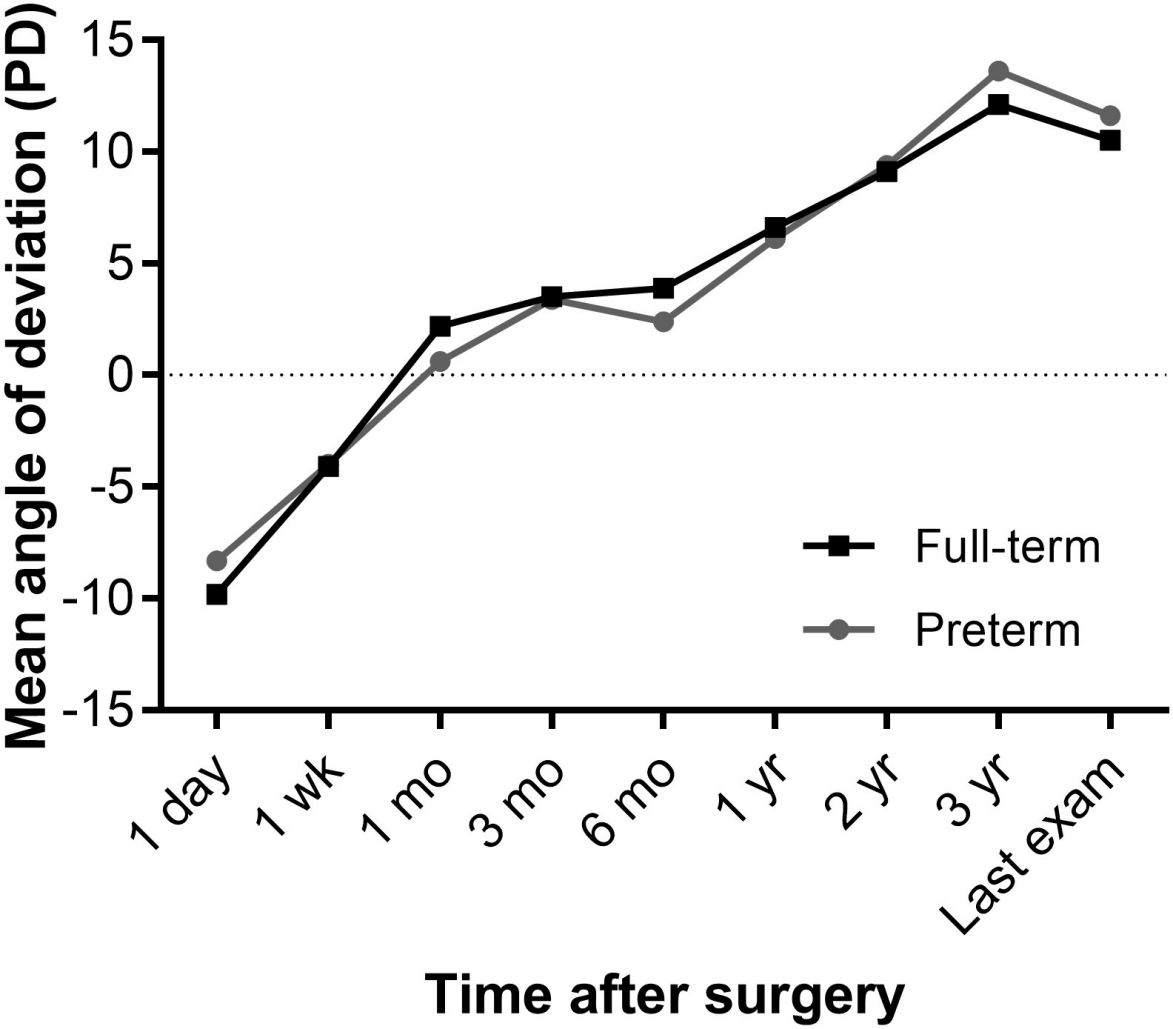
Postoperative angle of deviation according to postoperative duration between the pre-term and full-term groups. The effect of time on the angle of deviation from baseline (postoperative day 1) to final follow-up postoperatively was calculated using a linear mixed model analysis, which demonstrates a significant change in the angle of deviation over time (p<0.001). However, the postoperative angles of deviation between the two groups are not different (p = 0.734), and a group×time interaction effect is also not found (p = 0.992).

Table 3 provides the success results at 1 year and final follow-up visit after surgery in the pre- and full-term groups. The success, overcorrection, and recurrence rates between the pre- and full-term groups were not different at 1 year and final follow-up visit (p = 0.691 and 0.977, respectively). The major cause of surgical failure was recurrence in both groups. Postoperative ocular deviation after surgery is described in Fig 1. The angle of deviations over postoperative time using a linear mixed model showed no difference between the two groups (group × time interaction effect, p = 0.992).

**Table 3:** Surgical outcomes at 1 year and final follow-up visit after surgery in preterm and full-term groups.

		Preterm group (N = 48)	Full-term group (N = 432)	P-value^a^
1 year	Success	29 (60.4)	267 (61.8)	0.691
	Overcorrection	3 (6.3)	16 (3.7)	
	Recurrence	16 (33.3)	149 (34.5)	
Final visit	Success	21 (43.8)	191 (44.2)	0.977
	Overcorrection	1 (2.1)	11 (2.5)	
	Recurrence	26 (54.2)	230 (53.2)	

Data are presented as numbers (%).

^a^Pearson’s χ2 test

**Fig 2:**
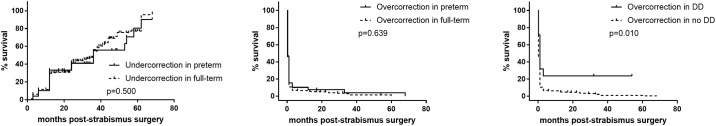
Survival curves for overall surgical recurrence and overcorrection results after surgery in exotropia. **(Left)** The recurrence rates are increased in both pre- and full-term children after surgery, and recurrence survival curves are similar between the pre- and full-term groups (p = 0.500). **(Middle)** The overcorrection survival curves are similar between the pre- and full-term groups (p = 0.639). **(Right)** Survival curves show a significantly larger cumulative overcorrection rate in patients with neurodevelopmental disabilities than in those without neurodevelopmental disabilities (p = 0.010).

The survival curves for overcorrection and recurrence rates in the pre- and full-term children are displayed in Fig 2. Analyses for overcorrection and recurrence showed no differences in both rates between pre- and full-term children (p = 0.639 and 0.500, respectively; log rank test). The recurrence rates increased in both pre- and full-term children after surgery.

### Risk factors of recurrence and overcorrection

The Cox proportional hazards regression model for the competing risk analysis for recurrence is displayed in Table 4. Pre-term birth is not a risk factor for recurrence. In the univariable regression analysis, younger age at onset of exotropia, absence of neurodevelopmental disabilities, intermittent exotropia, smaller preoperative ocular deviation at distance and near fixation, smaller amount of recession, and smaller immediately postoperative esodeviation were significantly associated with recurrence (p = 0.030, 0.018, 0.028, 0.019, 0007, 0.011, and 0.002, respectively). In the multivariable analysis, younger age at onset of exotropia and smaller immediately postoperative esodeviation significantly affected the recurrence rates (p = 0.001 and <0.001, respectively).

**Table 4:** Univariable and multivariable regression analyses for competing risk factors for overcorrection and recurrence.

Factors	Recurrence				Overcorrection			
	Univariable analysis		Multivariable analysis		Univariable analysis		Multivariable analysis	
	HR (95% CI)	P-value	HR (95% CI)	P-value	OR (95% CI)	P-value	OR (95% CI)	P-value
Age								
**onset of exotropia**	**0.994** **(0.988 to 0.999)**	**0.030**	**0.989** **(0.983 to 0.996)**	**0.001**	0.988 (0.956 to 1.021)	0.471		
diagnosis of exotropia	1.000 (0.996 to 1.004)	0.992			0.996 (0.975 to 1.016)	0.674		
**surgery**	1.000 (0.996 to 1.004)	1.000			**0.970** **(0.944 to 0.997)**	**0.028**	0.990 (0.966 to 1.015)	0.419
Preterm birth	1.139 (0.757 to 1.714)	0.532			1.228 (0.155 to 9.724)	0.846		
Low birth weight	1.250 (0.791 to 1.975)	0.339			0.298 (0.078 to 1.143)	0.077		
**Neurodevelopmental disabilities**	**0.302** **(0.112 to 0.813)**	**0.018**	0.483 (0.151 to 1.550)	0.221	**17.500** **(4.671 to 65.562)**	**<0.001**	**16.218** **(2.396 to 109.799)**	**0.004**
Sex (M:F)		0.167				0.198		
Male	1.000				1.000			
Female	0.841 (0.658 to 1.075)				2.379 (0.636 to 8.901)			
Refractive errors	1.031 (0.946 to 1.124)	0.481			1.003 (0.674 to 1.494)	0.987		
**Type of exotropia**		**0.028**	0.754 (0.547 to 1.039)	0.084		**0.006**		0.361
Intermittent	1.000				1.000		1	
Constant	0.723 (0.542 to 0.966)				5.472 (1.619 to 18.490)		2.039 (0.442 to 9.409)	
Preoperative deviation								
**Distance**	**0.980** **(0.963 to 0.997)**	**0.019**	0.979 (0.935 to 1.024)	0.348	**1.163** **(1.085 to 1.246)**	**<0.001**	1.027 (0.841 to 1.255)	0.792
**Near**	**0.978** **(0.962 to 0.994)**	**0.007**	0.999 (0.968 to 1.032)	0.965	**1.142** **(1.066 to 1.224)**	**<0.001**	0.988 (0.844 to 1.180)	0.982
Presence of eye dominance	1.201 (0.937 to 1.539)	0.149			0.730 (0.217 to 2.459)	0.612		
Lateral incomitance	0.808 (0.612 to 1.065)	0.131			2.427 (0.598 to 9.851)	0.215		
**Associated vertical strabismus**	1.035 (0.793 to 1.350)	0.800			**3.237** **(1.010 to 10.372)**	**0.048**	3.442 (0.804 to 14.736)	0.096
BLR:ULR recession procedure		0.267				0.381		
BLR	1.000				1.000			
ULR	0.833 (0.603 to 1.150)				0.398 (0.051 to 3.125)			
**Amount of recession**	**0.938** **(0.894 to 0.985)**	**0.011**	1.023 (0.909 to 1.151)	0.709	**1.565** **(1.266 to 1.935)**	**<0.001**	1.447 (0.738 to 2.836)	0.282
**Postoperative deviation**	**1.037** **(1.014 to 1.060)**	**0.002**	**1.050** **(1.024 to 1.077)**	**<0.001**	**0.829** **(0.742 to 0.925)**	**0.001**	**0.812** **(0.714 to 0.925)**	**0.002**
Binocular fusion at distance		0.850				0.793		
Fusion	1.000				1.000			
Nonfusion	1.029 (0.769 to 1.376)				0.786 (0.130 to 4.761)			
Binocular fusion at near		0.686				0.612		
Fusion	1.000				1.000			
Nonfusion	1.061 (0.796 to 1.415)				0.566 (0.063 to 5.115)			
Stereoacuity		0.631				0.365		
Good	1.000				1.000			
Bad	0.934 (0.708 to 1.233)				2.299 (0.380 to 13.922)			

HR = hazard ratio, OR = Odds ratio

The logistic regression model for the competing risk analysis for final overcorrection is displayed in Table 4. Pre-term birth is also not a risk factor for overcorrection. In the univariable regression analysis, younger age at surgery of exotropia, presence of neurodevelopmental disabilities, constant exotropia, greater preoperative ocular deviation at distance and near fixation, greater amount of recession, presence of associated vertical strabismus, and greater immediately postoperative esodeviation were significantly associated with final overcorrection (p = 0.028, <0.001, 0.006, <0.001, <0.001, 0048, <0.001, and 0.001, respectively). In the multivariable analysis, presence of neurodevelopmental disabilities and greater immediately postoperative esodeviation significantly affected final overcorrection (p = 0.004 and 0.002, respectively). Survival curves showed significant differences in the overcorrection rate according to the presence of neurodevelopmental disabilities (p = 0.010) (Fig 2).

### Sensory fusion and stereoacuity

Stereoacuity was measured in 390 patients (81%) during the preoperative examination and 395 patients (82%) during the postoperative examination. Worth 4-dot measurements were available from 378 and 390 patients (79% to 81%) during the pre- and postoperative examinations, respectively (Table 5). The most recent postoperative tests were performed at 20.1 ± 13.8 and 20.9 ± 14.8 months after surgery in pre- and full-term patients, respectively.

**Table 5:** Pre- and postoperative fusion and stereoacuity.

	Birth history		P-value	Neuro-developmental disabilities		P-value
	Preterm	Full-term		Yes	No	
Preoperative exam						
Stereoacuity (≤ 100 s of arc:> 100 s of arc)	23:14	212:141	0.803^a^	1:6	234:149	**0.017**^b^
Binocular fusion (Fusion:Nonfusion)						
Distance	14:21	120:234	0.469^a^	1:7	133:248	0.272^b^
Near	22:13	249:106	0.372^a^	1:7	270:112	**0.001**^b^
Postoperative exam						
Stereoacuity (≤ 100 s of arc:> 100 s of arc)	27:11	264:93	0.700^a^	1:7	290:97	**<0.001**^b^
Binocular fusion (Fusion:Nonfusion)						
Distance	20:18	178:165	0.931^a^	2:5	196:178	0.268^b^
Near	30:8	274:66	0.809^a^	3:4	301:70	**0.030**^a^

Data are presented as numbers.

^a^Pearson’s χ2 test

^b^Fisher’s exact test

Binocular fusion at distance and near, and stereoacuity showed no difference in pre- and postoperative examinations between the pre- and full-term groups (Table 5). However, pre- and postoperative stereoacuity was significantly poorer in patients with neurodevelopmental disabilities than in those without neurodevelopmental disabilities (p = 0.017 and <0.001, respectively). Pre- and postoperative binocular fusion at near also showed lower fusion in patients with neurodevelopmental disabilities than in those without neurodevelopmental disabilities (p = 0.001 and 0.030, respectively). Pre- and postoperative binocular fusions at distance were not significantly different between the two groups.

## Discussion

In this study, we compared the surgical outcomes of exotropic patients with pre- and full-term births. Preoperative ophthalmological characteristics were not significantly different between the pre- and full-term groups. Surgical success, overcorrection, and recurrence rates showed similar results between the two groups. The major cause of surgical failure was recurrence in both groups.

The cause of high prevalence of strabismus in prematurity might be ocular pathologies, including refractive error, retinopathy, and amblyopia or presence of cerebral damage. [1–4, 6, 18] It has also been suggested that premature children without any ocular or cerebral damage were at risk of visual impairment. [21] However, several studies reported that premature children without ocular and cerebral pathologies did not differ from full-term children in visual acuity or binocular vision and visual impairment in prematurity linked to the presence of cerebral pathologies. [22, 23] In our study, preoperative ocular characteristics were not different between the pre- and full-term groups, and pre-term birth was not a risk factor for recurrence or overcorrection. However, presence of neurodevelopmental disabilities was significantly higher in the pre-term group, which affected the final overcorrection. Sensory fusion and stereoacuity were also poorer in patients with neurodevelopmental disabilities than in those without neurodevelopmental disabilities. As subcortical white matter brain injuries are more frequent in immature infants, they probably contribute to the surgical outcomes. [24]

Pre-term birth is reported in 30%–60% of early onset esotropic cases, and a high rate of late overcorrection has been consistently reported in pre-term or neurologically impaired patients with esotropia. [5, 7, 12, 13] Surgery to correct esotropia is often delayed in this group of children, and the delay due to instability of angle measurements and poor cooperation has been suggested as a reason for the poor prognosis. [10, 11] The defective substrate of binocularity may be the reason for the high incidence of strabismus and unpredictable surgical outcomes in patients with CP. [25] A study on the surgical outcome of exotropia in premature children reported these children had a low surgical success rate, and the major cause of the surgical failure was recurrence. [18] Unlike the high rate of overcorrection in esotropic CP patients, studies reported that satisfactory ocular alignment in exotropic patients with CP could be achieved after BLR recession using the same amount of recession compared to age-matched exotropic patients without CP. [16, 17] In those studies, the main cause of surgical failure was recurrence. Han et al. reported that the CP group showed better surgical success (76.7%) and slower exotropic drift than the control group (56.7%). [16] Ma et al. reported that exotropic patients with CP showed a similar amount of exodrift and recurrence compared with the control group. [17] Postoperative exodrift of ocular alignment is also a common phenomenon in exotropic patients with CP, and this might explain the similar surgical responses in both groups. [17] They also reported that exotropic patients with CP resulted in a higher final overcorrection rate (12.2%) after BLR recession compared to those without CP (2.4%), although that was regarded as clinically insignificant. [17]

Our study demonstrated that the presence of neurodevelopmental disabilities was a risk factor for final overcorrection despite decreasing surgical dosing in children with those disabilities. The poor binocularity manifested by these patients is similar to the results of previous studies on esotropic patients with neurological impairments. Large overcorrection should be avoided because wearing spectacles could be difficult in patients with neurodevelopmental disabilities. [17]

The factors reported to affect the surgical outcome after exotropia surgery vary widely, including age at surgery, preoperative angle of deviation, refractive errors, type of surgery, lateral incomitance and early postoperative ocular alignment. [6, 19, 26–29] In this study, younger age at onset of exotropia and smaller amount of postoperative overcorrection were associated with recurrence, and presence of neurodevelopmental disabilities and greater amount of postoperative overcorrection were risk factors for final overcorrection in the multivariable analyses. Kim et al. reported that younger age at diagnosis and surgery and early postoperative overcorrection of ≥ 20 PD were predisposing factors for consecutive esotropia. [29]

Our study has some limitations that are mainly related to its retrospective study design. Most of the pre-term patients were not very pre-term, and the number of patients with ocular abnormalities associated with prematurity except exotropia were small. However, Petrini et al. reported that risk of neurodevelopmental consequences increases even in infants born at moderate prematurity (34 to 36 weeks of gestation). [8] Follow-up periods were not standardized. The strength of our study was the evaluation of stereoacuity and fusional status, although these were not available for patients with young age, poor cooperation, or lack of documentation.

In conclusion, pre-term patients with exotropia showed comparable surgical results to that of controls. Pre-term birth is not a risk factor for overcorrection and recurrence. Regardless of the pre- or full-term status, the presence of neurodevelopmental disabilities was a risk factor for final overcorrection.

## Supporting information

S1 File (XLSX)
